# Lumbar Interbody Fusion Conducted on a Porcine Model with a Bioresorbable Ceramic/Biopolymer Hybrid Implant Enriched with Hyperstable Fibroblast Growth Factor 2

**DOI:** 10.3390/biomedicines9070733

**Published:** 2021-06-25

**Authors:** Milan Krticka, Ladislav Planka, Lucy Vojtova, Vladimir Nekuda, Premysl Stastny, Radek Sedlacek, Adam Brinek, Michaela Kavkova, Eduard Gopfert, Vera Hedvicakova, Michala Rampichova, Leos Kren, Kvetoslava Liskova, Daniel Ira, Jana Dorazilová, Tomas Suchy, Tomas Zikmund, Jozef Kaiser, David Stary, Martin Faldyna, Martin Trunec

**Affiliations:** 1 Trauma Surgery Department, Faculty of Medicine, Masaryk University and The University Hospital Brno, 625 00 Brno, Czech Republic; krticka.milan@fnbrno.cz (M.K.); nekuda.vladimir@fnbrno.cz (V.N.); ira.daniel@fnbrno.cz (D.I.); 2 Department of Paediatric Surgery, Orthopedics and Traumatology, Faculty of Medicine, Masaryk University and The University Hospital Brno, 662 63 Brno, Czech Republic; planka.ladislav@fnbrno.cz (L.P.); stary.david@fnbrno.cz (D.S.); 3 CEITEC-Central European Institute of Technology, Brno University of Technology, 612 00 Brno, Czech Republic; premysl.stastny@ceitec.vutbr.cz (P.S.); adam.brinek@ceitec.vutbr.cz (A.B.); michaela.kavkova@ceitec.vutbr.cz (M.K.); jana.dorazilova@ceitec.vutbr.cz (J.D.); tomas.zikmund@ceitec.vutbr.cz (T.Z.); jozef.kaiser@ceitec.vutbr.cz (J.K.); martin.trunec@ceitec.vutbr.cz (M.T.); 4 Department of Mechanics, Biomechanics and Mechatronics, Faculty of Mechanical Engineering, Czech Technical University in Prague, 160 00 Prague, Czech Republic; radek.sedlacek@fs.cvut.cz; 5 Veterinary Research Institute, 621 00 Brno, Czech Republic; gopfert@vri.cz (E.G.); faldyna@vri.cz (M.F.); 6 University Center for Energy Efficient Buildings, Czech Technical University in Prague, 273 43 Bustehrad, Czech Republic; vera.hedvicakova@iem.cas.cz (V.H.); michala.rampichova@iem.cas.cz (M.R.); 7 Department of Tissue Engineering, Institute of Experimental Medicine of the Czech Academy of Sciences, Videnska 1083, 142 20 Prague, Czech Republic; 8 Department of Pathology, Faculty of Medicine of Masaryk University and The University Hospital Brno, 625 00 Brno, Czech Republic; kren.leos@fnbrno.cz (L.K.); liskova.kvetoslava@fnbrno.cz (K.L.); 9 Department of Composites and Carbon Materials, Institute of Rock Structure and Mechanics, The Czech Academy of Sciences, 182 09 Prague, Czech Republic; suchyt@irsm.cas.cz

**Keywords:** micro-CT, biomechanics, histology, animal model, lumbar spinal fusion, tissue engineering, autograft, ceramic, collagen, FGF2

## Abstract

Many growth factors have been studied as additives accelerating lumbar fusion rates in different animal models. However, their low hydrolytic and thermal stability both in vitro and in vivo limits their workability and use. In the proposed work, a stabilized vasculogenic and prohealing fibroblast growth factor-2 (FGF2-STAB^®^) exhibiting a functional half-life in vitro at 37 °C more than 20 days was applied for lumbar fusion in combination with a bioresorbable scaffold on porcine models. An experimental animal study was designed to investigate the intervertebral fusion efficiency and safety of a bioresorbable ceramic/biopolymer hybrid implant enriched with FGF2-STAB^®^ in comparison with a tricortical bone autograft used as a gold standard. Twenty-four experimental pigs underwent L2/3 discectomy with implantation of either the tricortical iliac crest bone autograft or the bioresorbable hybrid implant (BHI) followed by lateral intervertebral fixation. The quality of spinal fusion was assessed by micro-computed tomography (micro-CT), biomechanical testing, and histological examination at both 8 and 16 weeks after the surgery. While 8 weeks after implantation, micro-CT analysis demonstrated similar fusion quality in both groups, in contrast, spines with BHI involving inorganic hydroxyapatite and tricalcium phosphate along with organic collagen, oxidized cellulose, and FGF2- STAB^®^ showed a significant increase in a fusion quality in comparison to the autograft group 16 weeks post-surgery (*p* = 0.023). Biomechanical testing revealed significantly higher stiffness of spines treated with the bioresorbable hybrid implant group compared to the autograft group (*p* < 0.05). Whilst histomorphological evaluation showed significant progression of new bone formation in the BHI group besides non-union and fibrocartilage tissue formed in the autograft group. Significant osteoinductive effects of BHI based on bioceramics, collagen, oxidized cellulose, and FGF2-STAB^®^ could improve outcomes in spinal fusion surgery and bone tissue regeneration.

## 1. Introduction

The use of lumbar fusion procedures in the USA and Europe has rapidly increased over the last decade, and many of these procedures involve the use of bone grafts [1]. Despite the technical progress of spinal surgery and operative materials, the risk of vertebral fusion failure occurs in 5–35% of cases [2]. Successful fusion depends on several surgical and host factors including the selection of a bone graft or bone substitute with adequate osteoconductive, osteoinductive, and osteogenic properties [3]. Autografting has been considered the gold standard for bone graft procedures as it poses little risk of infection and rejection. However, harvesting from the iliac crest can be associated with short- and long-term morbidity in up to 22% of cases [4]. Spinal fusion is a well-accepted surgical treatment for many spinal diseases with significant increases projected due to an ageing population. However, non-unions are a notable complication of spinal fusion surgery. The limitations have resulted in the utilization of synthetic alternative materials for bone repair, replacement, and enhancement [5].

Contemporary scaffolds of the third-generation try to get closer to autograft levels by using biomaterials modified by instructive substances (biological factors or external stimuli) capable of inducing specific beneficial biological responses mainly bone formation and vascularization [5]. Whereas the utilization of autologous mesenchymal stem cells (MSCs) involves various problems (in vitro cultivation, sterility, high cost, use of fetal bovine serum, time duration etc.) resulting in time-delayed second operation, a possible alternative could be the use of bioimplants enriched with growth factors acting in the cascade of fusion and fracture healing [6]. At present, the only tissue-engineered product involving a growth factor that is FDA-approved for spinal fusion is a collagen sponge delivered with recombinant human bone morphogenetic protein 2 (rhBMP-2) (INFUSE Bone Graft, Medtronic). However, this product is associated with significant complications, which are thought to arise from the supraphysiological therapeutic dose of rhBMP-2 required for effective bone formation [7]. In addition to rhBMP-2, recombinant human parathyroid hormone (rhPTH) and recombinant human BMP-7 (rhBMP-7) have been studied clinically in spinal fusion [8]. Among the identified growth factors, calcitonin, GDF-5, NELL-1, P-15, AB204, angiopoietin 1, insulin, and peptide B2A resulted in significantly enhanced fusion rates compared to iliac crest bone graft or other internal controls in some animal studies [9]. Besides, basic fibroblast growth factor (bFGF; FGF-β), also known as fibroblast growth factor 2 (FGF2), has a primary function in cartilage and bone targeted at morphogenesis, mineralization, and metabolism [10]. The use of FGF2 in spinal fusion was reported only by Inoue et al. that investigated the effects of the bone allograft incubated with engineered FGF2 in a Sprague-Dawley rat posterolateral lumbar (L4-L5) model. The FGF2 improved significantly mean grafted bone volume as well as the new bone formation on the surface of laminae and spinous processes compared to the bone allograft without FGF2 [11].

Based on the review above, FGF2 can be added to the implant as an osteoinductive substance accelerating osteogenesis, enhancing the proliferation of the osteoblast cell line as well as inducing angiogenesis [12]. However, applications of FGF2 protein are limited due to its low stability in vivo and in vitro [13]. Here we tackle this problem using a hyperstable FGF2-STAB^®^ being an isoform of FGF2 generalized by computer-assisted protein engineering strategy with in vitro functional half-life at 37 °C improved from 10 h to more than 20 days [14].

This study aims to extend our previous work provided on animal models using 3rd generation scaffolds. Previously, porous collagen scaffolds modified with either hydroxyapatite (HA) or tricalcium phosphate (TCP) and seeded with MSCs were applied to heal large segmental bone defects, bone bridges or hyaline cartilage [15,16,17,18]. The soft collagen network provides a favorite nanofeatured, high surface area environment, which can be easily degraded in weeks or months. This degradation opens the space for vascularization and further growth of bone cells, while the ceramic HA/TCP skeleton with controlled degradation offers sufficient support until new bone formation. Both collagen and hydroxyapatite scaffolds are the most used materials for bone regeneration [19,20]. They have excellent biocompatibility with hard tissues, high osteoconductivity, and bioactivity, and they are neither antigenic nor cytotoxic [21]. They enhance the bioactivity of the scaffold by providing a source of calcium and phosphate ions that can be used by osteogenic cells to create their own bone [22].

However, the clinical use of MSCs in combination with synthetic scaffolds might be difficult and cause a few problems as already mentioned above. To accelerate bone formation, MSCs can be exchanged, e.g., for platelet-rich plasma (PRP), since the excellent properties of PRP for tissue engineering and regenerative medicine have been revealed [23]. PRP can release multiple growth factors and play an important role in the complex local inflammatory response, promote angiogenesis, and recruit mesenchymal cells [24]. Recently, we found that the combination of collagen with natural polysaccharides like chitosan and oxidized cellulose exhibited a significant positive synergistic effect with platelet lysate on the cultivation of cells, as well as on angiogenesis evaluated by ex ovo Chick Chorioallantoic Membrane (CAM) assay comparing to pure collagenous scaffold enriched only with platelet lysate [25]. Moreover, hemostatic and antibacterial properties make both chitosan and oxidized cellulose good candidates for tissue engineering scaffolds, limiting the risk of infection [26]. Above that, oxidized cellulose possesses non-immunogenicity, anti-tumour activity, high absorbability, anti-adhesive effects, biocompatibility, and bioresorbability [27]. The bioresorption is enabled via chemical depolarization and enzymatic hydrolysis mediated by glycosidases, which leads to nontoxic final products of glucuronic acid and glucose [28]. Newly, we verified the safety, healing, and angiogenic potential of collagen-based scaffolds enriched with FGF2-STAB^®^ as full skin replacement in vivo on 3-month-old White New Zealand rabbits [29]. Biodegradable scaffolds revealed promising results in cell culture experiments and have displayed high suitability and biocompatibility to be used as a transferable scaffold for tissue reconstruction [30]. However, the aforementioned collagen-based scaffolds are very soft and elastic, being excellent for skin regeneration but lack structural stability upon hydration and mechanical strength, which limit their applications in particular bone tissues [31]. To eliminate the shortcoming, we have prepared sintered calcium phosphate scaffolds with different phase structures having similar cellular pore size and an open porosity of over 80%. The biphasic calcium phosphate scaffold (BCP) with the highly soluble α-TCP phase embedded in a less soluble matrix of β-TCP and HA exhibited a controllable degradation within an acidic environment simulating the osteoclastic activity. Moreover, a suitable strength, stability, and excellent cell response tested in vitro predestined the BCP to be used as resorbable load-bearing bone scaffolds [32].

In this work, a biomimetic third-generation bioresorbable hybrid implant based on the combination of sintered foamed BCP matrix filled with cross-linked collagen type I and oxidized cellulose assembled with FGF2-STAB^®^ has been utilized for an animal experimental study in a porcine model to compare its outcomes with an iliac crest autograft in generating solid lumbar interbody fusion.

## 2. Materials and Methods

### 2.1. Implant Preparation

The bioresorbable hybrid implant (BHI) based on the ceramic foam coated with biopolymers and FGF2-STAB^®^ was prepared in two steps. In the first step, a porous biphasic calcium phosphate scaffold (BCP) ceramic foam was fabricated via gel casting and sintering methods according to our previous works [32,33]. Briefly, HA powder (extra pure, Riedel-de Haen, Seelze, Germany) calcined at 1000 °C/3 h and a mixture of an α- and β-TCP powder (purum p.a, Honeywell, Seelze, Germany) were used to prepare an aqueous epoxy-based ceramic slurry. A ceramic foam, prepared by a direct foaming of the biphasic HA/TCP slurry, was cast into a mold with a diameter of 100 mm and a height of 10 mm and polymerized. After consolidation, the ceramic foam was dried, segmented in implants preforms, sintered at 1250 °C and milled to the final size of the implant (25 mm × 15 mm × 3.3 mm). The second step involved impregnation of the ceramic implants with cross-linked biopolymer fibers based on bovine collagen and calcium salt of oxidized cellulose assembled with hyperstable Fibroblast Growth Factor 2 (FGF2-STAB^®^). Shortly, type I collagen from bovine skin (Collado, Brno, Czech Republic) was disintegrated in MilliQ ultrapure water Type I (prepared according to ISO 3696 on Elix 5 UV Water Purification System, Merck, Darmstadt, Germany) together with calcium salt of oxidized cellulose (LIFE LINE plus, Brno, Czech Republic) in a ratio of 1:1 giving total concentration of 0.5 wt% according to [25]. The collagen/cellulose mixture was injected via a 20G needle into the ceramic foam to cover all free implant volume followed by freeze-drying at −35 °C under 1 mBar for 15h (lyophilizer Martin Christ Epsilon 2-10D, Martin Christ, Osterode am Harz, Germany) followed by a secondary drying process at 25 °C under 0.01 mBar until decreasing Δp (the change in pressure was up to 10%). To stabilize biopolymers and postpone the FGF2-STAB^®^ release, foams were treated with N-ethyl-N’-[3-dimethylaminopropyl] carbodimide/ N-hydroxy succinimide (EDC/NHS) (Sigma-Aldrich, Darmstadt, Germany) cross-linking system in a molar ratio of 2/1 as verified earlier [34]. After 2 h cross-linking process, the BHI was washed twice with 0.1 M disodium phosphate (Sigma-Aldrich, Darmstadt, Germany) followed by pure water for removal of byproducts. At the end, the cold (4 °C) water solution of FGF2-STAB^®^ (Enantis, Brno, Czech Republic) was poured on the BHI in the amount of 0.1 µg of protein FGF2-STAB^®^ per 1 cm^2^ of BHI and consequently freeze-dried again as described above. FGF2-STAB^®^ was prepared by computer-assisted engineering of a unique nine-point mutant of the low molecular weight isoform FGF2 [35]. Prior to biological testing, all scaffolds were sterilized by ethylenoxide standard procedure.

### 2.2. Scaffold Morphology

Scaffold morphology was investigated employing a scanning electron microscope (SEM, Tescan MIRA3, Brno, Czech Republic). All observations were made in the secondary electron emission mode with a high voltage of 10 kV. For better resolution, the scaffolds were coated with the 20 nm gold layer. Pore size of the prepared scaffolds was calculated from the SEM images using semi-automatized threshold method. (ImageJ, U. S. NHS, Bethesda, MD, USA) [36]. Total number of 69 pores were distinguished for BCP and number of 244 pores for BHI.

### 2.3. Mechanical Testing

Compression tests of BCP and BHI materials were performed according to the ISO 13314 standard on 10 cubic samples of each type with dimensions 5 × 5 × 10 mm. The samples were tested in the wet state after hydration for 60 min in PBS solution at 37 °C. Ultimate compression strength, elastic gradient, and energy absorption were determined using an MTS Mini Bionix 858.02 system (MTS, Eden Prairie, MN, USA) equipped with a 100 N load cell. The measurements were carried out at a constant crosshead speed of 3.0 mm/min (deformation rate approx. 0.005 s^−1^). The obtained stress-strain curves were used to determine the compressive strength, elastic gradient, and the energy absorbed during compression. The ultimate compression strength was calculated as the maximum compression force divided by the cross-sectional area of the tested specimen. The elastic gradient was calculated as the gradient of the elastic straight line determined by the elastic loading and unloading process of compression testing. Energy absorption was calculated as the area under the stress-strain curve up to 40% strain. The elastic gradient used in the study represents the closest concept to that of Young’s modulus, which is determined for solid materials. To simplify the comparison of our results and the results of other studies, we assumed that the elastic gradient represents the modulus of elasticity under compression.

### 2.4. In Vitro Testing of Ceramic-Based Scaffolds

Both pure ceramic scaffolds and ceramic/biopolymer hybrid scaffolds were tested in view of cytotoxicity. Scaffolds with a size of 5 × 5 × 3 mm were placed in 96-well plates, prewet with 150 µL of differentiation medium (α-MEM supplemented with 10% fetal bovine serum (FBS), Penicillin/Streptomycin (100 IU/mL and 100 μg/mL), 100 nM dexamethasone, 10 mM β-glycerol phosphate and 50 µg/mL ascorbate-2-phosphate). Scaffolds were seeded with 85 × 10^3^ human bone marrow-derived mesenchymal stem cells (hMSCs; ScienCell, Karlsbad, CA, USA) per well. Scaffolds were further placed in lysis buffer and the amount of DNA was measured using Quant-iT™ dsDNA Assay Kit (Life Technologies, MA, Waltham, MA, USA) according to the manufacturer manual. Confocal microscopy (LSM DUO, Zeiss, Germany, Oberkochen) was used to visualize cell adhesion and distribution on the scaffolds. Samples were stained using fluorescent probe 3,3‘-diethyloxacarbocyanine iodide (DiOC6(3), Invitrogen, 1 μg/mL in PBS) and propidium iodide (PI; 5 μg/mL in PBS).

### 2.5. Animal Model and Study Design

Twenty-four castrated male clinically healthy large white pigs, 4 months old, weighing around 40 kg were supplied by a local production company approved by Ministry of Agriculture of the Czech Republic and housed at the Veterinary Research Institute (Brno, Czech Republic) in experimental stables certified by Ministry of Agriculture of the Czech Republic. The study was conducted according to the guidelines of the Declaration of Helsinki and approved by the Institutional Review Board of Veterinary Research Institute (protocol code 21/2016 with approval from 24 March 2017) and by the Branch Commission for Animal Welfare of the Ministry of Agriculture of the Czech Republic (permission number 21395/2017-MZE-17214 from 4 April 2017). All pigs, divided into two study groups depending on the fusion method, underwent lateral lumbar interbody fusion (L2/3) either with implantation of an iliac crest bone graft (group A, *n* = *12*) or with a biomimetic BHI (group B, *n* = *12*). Twelve animals were sacrificed at week 8 (subgroup A1, *n* = *6* and B1, n = 6), whereas the remaining 12 were sacrificed at week 16 (subgroup A2, *n* = *6* and B2, *n* = *6*). Plain radiographs were regularly repeated after surgery. The spine interval from T15 to L6 was harvested en bloc and cleaned from the surrounding tissues while preserving the ligamentous stabilizers. Subsequently, implant removal and macroscopic inspection of the spine interval was performed. As controls, 7 cadaveric intact lumbar spines (group N, *n* = *7*) were used. All resected porcine spines from group A (*n* = *12*); group B (*n* = *12*) and group N (*n* = *7*) were dipped in 40% formaldehyde water solution and sealed into the plastic foil to eliminate the autolysis and drying of the sample.

### 2.6. Surgical Method

Animals were premedicated and anesthetized intramuscularly with Tiletamine 2 mg/kg + Zolazepam 2 mg/kg (Zoletil, Virbac, Prague, Czech Republic), Ketamin 2 mg/kg (Narketan, Vetoquinol, Paris, France) and Xylazin 2 mg/kg (Sedazine, Fort Dodge Animal Health, Fort Dodge, IO, USA). Endotracheal intubation was performed and mechanical ventilation with air-oxygen mixture (60/40) was used during total intravenous anesthesia. Intravenous anesthesia was maintained by Propofol infusion—10 mL/h (Propofol 1%, Fresenius Kabi, Bad Homburg, Germany). Prophylactic antibiotic treatment—Amoxicillin/clavulanic acid 15 mg/kg (Amoksiklav, Lek Pharmaceuticals, Ljubljana, Slovenia) was administered once before operation. After routine preparation and draping, L2/3 space was fluoroscopically identified and a left mini-lumbotomy was performed. L2/3 vertebras were visualized via extra-peritoneal approach. The intervertebral disc of interest was exposed after mobilization of the psoas muscle. Under fluoroscopy control, guiding lateral plates (Anterior Spinal Srew-rod System, Beijing Fule Science and Technology Development, Beijing, China) were implanted from the lateral side to L2 and L3 vertebrae. Each plate was fixed with two mono-axial pedicle screws—5 × 30 mm and 5 × 45 mm (Anterior Spinal Srew-rod System, Beijing Fule Science and Technology Development, Beijing, China). Further L2/3 discectomy was performed. The endplates were shaved using a curette down to the bleeding bone. Iliac crest bone graft was harvested from the left iliac bone and after distraction of L2/3 space, the tricortical bone graft was inserted press-fit into the intervertebral space (Group A, *n* = 12). Alternatively, the optimal size of biomimetic BHI was “press-fit” inserted into the empty intervertebral space (Group B, *n* = 12). Inserted pedicle screws were connected by 2 titanium rods (Anterior Spinal Srew-rod System, Beijing Fule Science and Technology Development, Beijing, China) to maintain the position and stability of the operated vertebral segment at the end of the surgical procedure. Each wound was irrigated with saline, the subcutaneous tissue and skin were approximated using interrupted sutures, and the skin was covered with Chlortetracycline spray (Pederipra, Hipra, Girona, Spain). Before the pig recovered from anesthesia, conventional lateral and anteroposterior radiographs were made.

### 2.7. General Observation, X-ray Imaging

After surgery, pigs were checked until full recovery from anesthesia. Two units of Amoxicillin/clavulanic acid 15 mg/kg (Amoksiklav, Lek Pharmaceuticals, Ljubljana, Slovenia) were administered every 72 h for one week. Pigs received Ketoprofen analgesics (Comforion Vet, Orion Pharma Animal Health, Espoo, Finland) intramuscularly regularly for one week after surgical procedure.

Pigs were housed individually with free access to water. General and neurological examinations were performed daily during the first week and twice per week later. Plain radiographs of antero-posterior and lateral views were regularly repeated after 2, 4, and 8 weeks after surgery in group A. In group B X–rays were taken after 2, 4, 8, 12, and 16 weeks. Pigs in both groups were euthanized by i.v. administration of Embutramide, Mebezonium iodide, Tetracaine hydrochloride injectable solution at 6ml/50kg (T61, MSD Animal Health, Kenilworth, NJ, USA).

### 2.8. X-ray Computed Microtomography (Micro-CT)

All resected porcine spines from group A *(n* = 12) and group B (*n* = 12) were subjected to the micro-CT analysis performed the same day as animal euthanasia. The micro-CT system GE phoenix v|tome|x L 240 (GE Sensing & Inspection Technologies GmbH, Wunstorf, Germany) equipped with Nanofocus180 kV/15 W X-ray tube and flat-panel detector DXR 250 was used for the tomographic measurement. The X-ray tube was set at an accelerating voltage of 100 kV and a current of 300 µA. The X-ray spectrum was filtered by a 1.5 mm thick aluminum filter. The detector exposure time was 400 ms in each of the 2200 positions. The measurement was performed at a constant temperature of 21 °C. The voxel size was 40 μm. The tomographic reconstruction was realized by GE phoenix datos|x 2.0 with a sample drift correction, beam hardening correction, and noise filtration. The registration of the vertebras according to the top-cranial and bottom-caudalis (ventralis, dorsalis) of the sample was conducted in VG Studio MAX 3.1 (Volume Graphics GmbH, Heidelberg, Germany). The registration simplifies the analysis of LIF. The bone graft lies in the same reconstructed area and has the same orientation.

### 2.9. Biomechanical Testing

**Figure 1:**
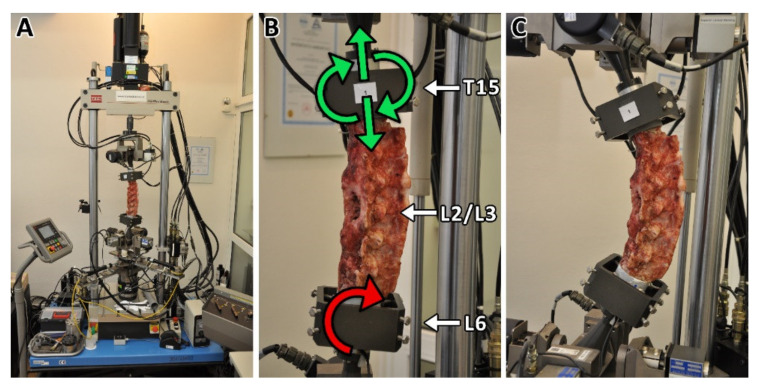
The MTS Bionix Spine Kinematics System (**A**) with fixed porcine spine cadaver. The inferior end of the lumbar spine cadaver (L6) was rigidly mounted on the platform on which the pure bending moment was applied (**B**). The superior end (T15) was unconstrained and free to move in the vertical direction and to rotate (**B**). The composite implants or bone graft were implanted from the lateral side between L2 and L3 vertebras. Pure bending moment was applied with a load limit of 5 Nm at a rotation rate of 20°/min in flexion (+40°)—extension (−40°) mode (**C**). Each specimen was tested for three cycles.

Biomechanical evaluation of lumbar spinal stability after surgical intervertebral fusion was carried out on the MTS Bionix Spine Kinematics System 370.02 platform (MTS, Eden Prairie, MN, USA). Testing was carried out on the following day after animal euthanasia. The flexural stiffness in extension of the lumbar spine cadavers (T15-L6) free of all soft tissues except for intervertebral ligaments and facet joint capsules were evaluated, namely the native specimens (*n* = 7), group A (A1, *n* = 4; A2, *n* = 4) and group B (B1, *n* = *4*; B2, *n* = 4). The six-axis force-moment sensor Mini 45 SI-580-20 (MTS, Eden Prairie, MN, USA) was used for load measurement. The cadavers were anchored to the testing system (see Figure 1) and a pure bending moment was applied to the rigidly mounted inferior end (L6). The superior end (T15) was unconstrained, free to move in a vertical direction and rotate. In the non-destructive mode, 3 cycles of pure bending moments were applied (5 N·m load limit) at a rate of 20°/min in flexion (+40°) and extension (−40°). A total of 87 load records were processed and subsequently evaluated. From the obtained graphs of individual group samples, one representative sample having average stiffness was selected.

### 2.10. Histological Evaluation

To conduct a scientific and accurate histomorphological analysis, 5 representative histological sections of the intervertebral segment in the sagittal plane were obtained from 8 samples, group A (A1, *n* = 2; A2, *n* = 2) and group B (B1, *n* = *2*; B2, *n* = *2*). Evaluation and photographic documentation were performed with an Olympus BX45 microscope (Olympus Optical, Tokyo, Japan). Olympus Viewfinder Lite™ software was used to acquire and process the images. The sections with the largest area of newly formed bone tissue were scanned with a Axio Scan Z1 Digital Slide Scanner (Carl Zeiss Microscopy GmbH, Göttingen, Germany) with a plan apochromatic objective with a magnification of 20×/0.8 M27. The areas of mature bone were exactly quantified (in mm^2^) using the Imaging Software ZEN 2.6 (blue edition, Carl Zeiss Microscopy GmbH, Göttingen, Germany). To evaluate the relative content (%) of the newly formed trabecular bone values from the whole implantation area of intervertebral fusion, an ImageJ software (Public Domain, Java-based image processing program developed at the National Institutes of Health and the Laboratory for Optical and Computational Instrumentation at University of Wisconsin) was applied.

### 2.11. Statistical Analysis

Data normality were analyzed via Shapiro–Wilk test. Statistical analysis was performed using statistical software (STATGRAPHICS Centurion XVII, StatPoint, Warrenton, VA, USA). Non-parametric analysis was employed since either the assumptions of normality or homoscedasticity were violated and, subsequently, the Kruskal-Wallis test for multiple comparisons with the subsequent post hoc test based on the Bonferroni procedure. The nonparametric Mann–Whitney test was applied for a pairwise comparison of (i) any of the treated groups and the native cadavers, (ii) the time points of each of the A and B groups, (iii) the A and B group at each of the time points. Statistical significance was accepted at *p* ≤ 0.05.

## 3. Results

### 3.1. Bioresorbable Hybrid Implant Properties

The composition of the ceramic matrix used in this work has been evaluated in terms of its chemical structure, biodegradation, mechanical properties, and morphology [32]. The final composition of the ceramic matrix examined by X-ray powder diffraction confirmed a biphasic calcium phosphate (BCP) comprised of hydroxyapatite (26%) and TCP involving β-TCP (62%), and α-TCP (12%). The BCP sample (having open porosity of 85%) has been chosen for this work according to its optimal degradation rate, being slower than that of the pure β-TCP but faster than that of the stable pure hydroxyapatite. The degradation of BCP in an acidic environment simulating osteoclastic activity (pH = 5.5) reached almost one-third the value of the strength in compression at day 14 of full hydration in comparison to the hydrolytically stable hydroxyapatite exhibiting no statistically significant effect of acidic environment on its stability. Therefore, the BCP should be fully resorbable in vivo within the preclinical testing on a porcine animal model.

**Figure 2:**
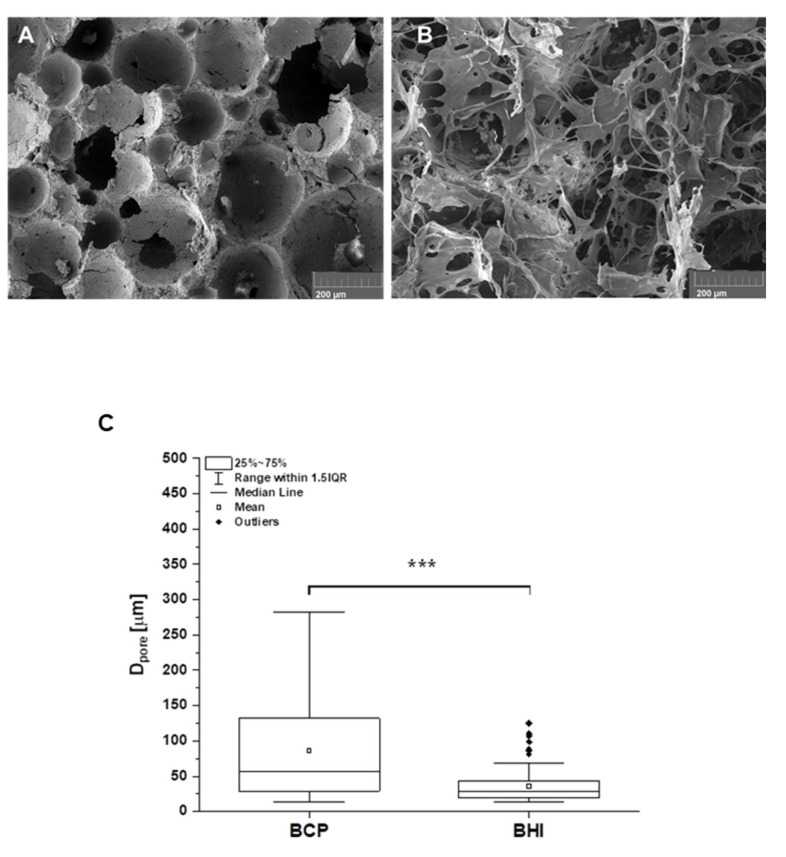
SEM micrographs of the structure of the pure biphasic calcium phosphate scaffold (BCP) (**A**) and the ceramic matrix impregnated with collagen, oxycellulose, and FGF2-STAB^®^ cross-linked network—bioresorbable hybrid implant (BHI) (**B**). Scale bar is 200 µm. Figure (**C**) represents the pore size distribution calculated from SEM images (**A**) for BCP (*n* = 69 of measured values) and (**B**) for BHI (n = 244 of measured values) using ImageJ program. Statistical significance *** *p* ≤ 0.001 (Mann–Whitney test).

The morphology of the original pure inorganic BCP foam matrix and that one modified with organic collagen type I, oxidized cellulose, and FGF2-STAB^®^ cross-linked organic network, stated as BHI, is shown in Figure 2A,B, respectively. As calculated from the SEM images, BCP foam had spherical interconnected pores with an average pore size of 86 µm (Figure 2C). After the impregnation of the ceramic foam with the network of cross-linked nanostructured biopolymer fibers filled the pores and covered the surface of the implant with bioactive polymers (Figure 2B). The porosity has significantly decreased down to pore size with an averaged value of 36 µm while keeping the open-pore structure of BHI scaffold (Figure 2C).

**Figure 3:**
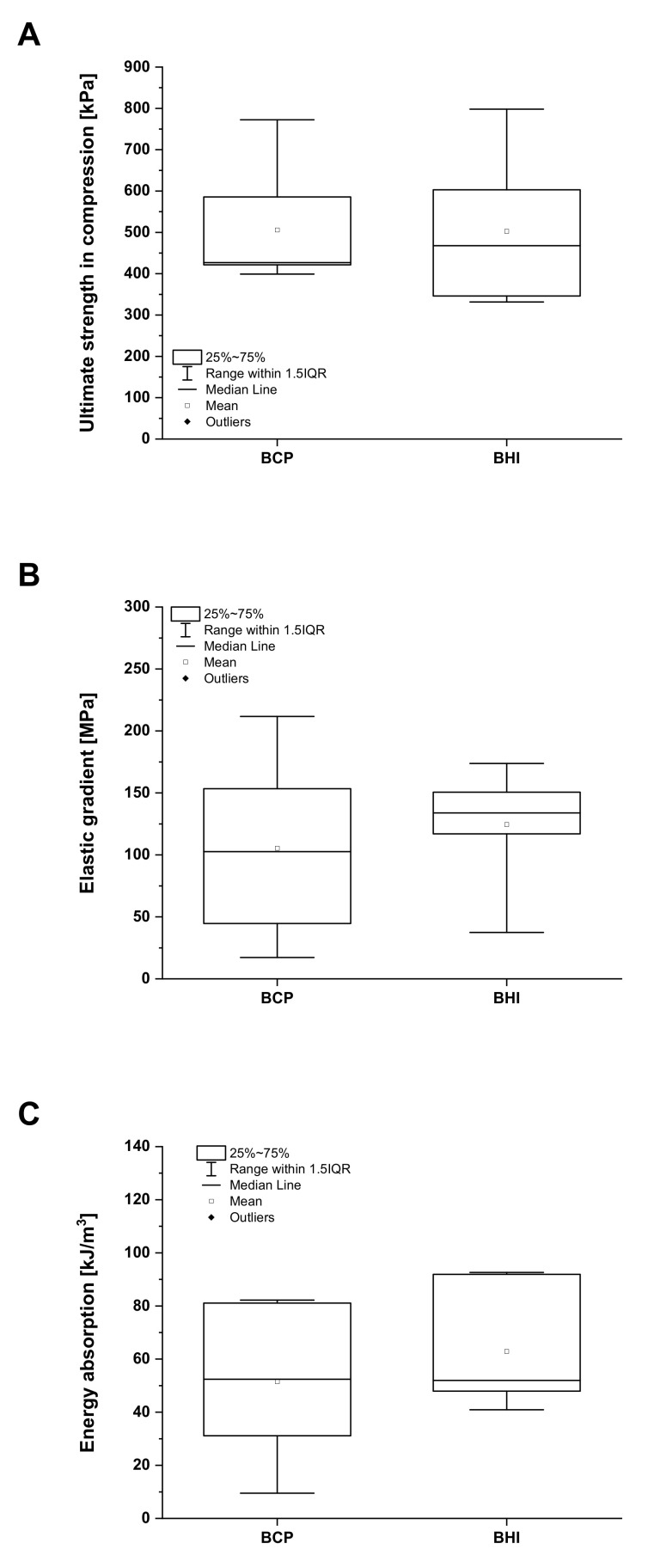
Mechanical properties of fully hydrated biphasic calcium phosphate scaffold (BCP) and bioresorbable hybrid implant (BHI). Ultimate strength in compression (**A**), elastic gradient (**B**), and energy absorption (**C**). No statistical significance (Mann–Whitney test, *p* < 0.05).

Both the BCP and BHI materials have been characterized in terms of their mechanical properties after hydration for 60 min in PBS solution at 37 °C. The results obtained from the mechanical tests show no statistically significant differences (*p* = 0.05). The ultimate compression strength for BCP and BHI after full hydration was very similar, equal to 0.51 ± 0.15 MPa and 0.50 ± 0.18 MPa, respectively, not showing any effect of biopolymeric impregnation (Figure 3A). However, the polymeric network positively affected the modulus of elasticity in compression that corresponded to 105 ± 76 MPa and 124 ± 47 MPa for BCP and BHI, respectively (Figure 3B). Consequently, the biopolymeric network increased the energy absorption (the total of absorbed energy per unit volume) from 51 ± 29 kJ/m^3^ for BCP to 63 ± 23 kJ/m^3^ for BHI (Figure 3C). During energy absorption, the materials need to be able to maintain a gradual deterioration in the load profile, which is important for load-bearing intervertebral fusion materials.

### 3.2. Cytotoxicity of Prepared Scaffolds

Recently, BCP showed superior biocompatibility features based on the evaluation of hMSCs metabolic activity, adhesion, and proliferation within two weeks cultured in comparison to the pure HA and β-TCP scaffolds [32]. Therefore, in the present study, we used the same BCP 3D matrix as a control group for 21 days cytotoxicity evaluation of the BCP matrix impregnated with collagen, oxidized cellulose, and FGF2-STAB^®^ (BHI). The amount of FGF2-STAB^®^ protein has been widely evaluated in our previous studies [29,30]. Based on these results, the concentration equal to 0.1 µg/cm^2^ has been chosen as the safe and effective one.

**Figure 4:**
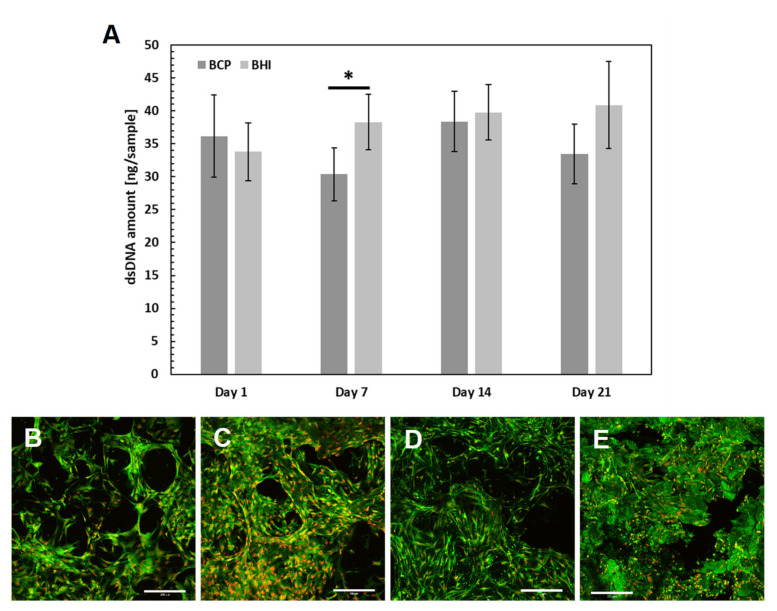
In vitro verification of ceramic implants biocompatibility: hMSC proliferation was measured using dsDNA quantification (**A**). Statistical significance is shown by bars above the columns (*p* < 0.05). Visualization of cell adhesion and distribution on scaffolds using a confocal microscope. Biphasic calcium phosphate scaffold (BCP) on day 1 (**B**), bioresorbable hybrid implant (BHI) implant on day 1 (**C**), BCP implant on day 14 (**D**), BHI implant on day 14 (**E**). Cell nuclei were stained using propidium iodide (red color) and intracellular membranes using DiOC6(3) (green color), scale bar 200 μm. Abbreviations: hMSC, human mesenchymal stem cells; BCP, pure ceramic implant; BHI, ceramic implant with biopolymers and FGF2-STAB^®^.

The number of hMSCs measured using cell DNA quantification was comparable on both BCP and BHI scaffolds within the whole experiment, except day 7, when BHI exhibited significantly higher DNA amount (Figure 4A) probably because of FGF2-STAB^®^ protein effect. As confirmed by confocal microscope images, the cells spread over the scaffold within 24 h and during the 21 days of the experiment formed confluent layers on both implants (Figure 4B–E).

Therefore, no cytotoxicity of the ceramic implant after biopolymer enrichment was detected, and the biomimetic hybrid implant was found to be biocompatible. However, the complexity of the tissue, e.g., the interaction of different cell types and signaling, is missing. Therefore, the hybrid implant was further tested in vivo to evaluate the osteoinductive potential of BHI modified by FGF2-STAB^®^ in the complex environment of bone tissue.

### 3.3. Surgery

**Figure 5:**
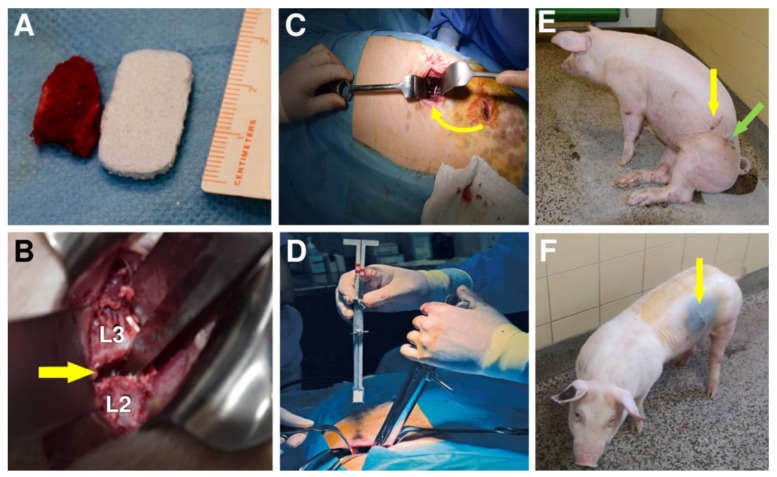
Photograph comparing bone autograft with bioresorbable hybrid implant (BHI) (**A**). Vertebrae L2 and L3 of the lumbar spine, between which the implant was placed (**B**). Iliac crest bone graft implantation (Group A) into L2/L3 segment indicated by an arrow (**C**). Implantation of BHI into L2/L3 segment in Group B using a special implant holder (**D**). A pig after autografting surgery (**E**). Green arrow shows the place where the autograft was withdrawn and yellow arrows the place of implantation. Last photo (**F**) shows a pig with one scar (yellow arrow) after the BHI implantation (Group B).

Twenty-four pigs enrolled in our study underwent lateral lumbar interbody fusion (L2/3, Figure 5B) either with implantation of an iliac crest bone graft (Figure 5C, Group A, *n* = *12*) or with a biomimetic BHI (Figure 5D, Group B, *n* = *12*). Twelve animals were sacrificed at 8 weeks (Subgroups A1, *n* = *6* and B1, *n* = *6)*, whereas the remaining 12 were sacrificed at a week after 16 weeks (Subgroup A2, *n* = *6* and B2, *n* = *6*). It is clear from Figure 5A that the BHI could be prepared in dimensions that matched the surgical area more appropriately than the autograft.

All experimental animals underwent surgery without major complications. However, the removal of a bone graft from the coxal tuber of the hip bone is associated with significantly longer operating time in group A (mean operating time = 77 min), compared to group B (45 min), higher blood loss during surgery in group A (52 mL) than in group B (32 mL) and slower walking ability after the surgery (648 min in average) (Figure 5E), comparing to only 80 min in group B (Figure 5F).

**Figure 6:**
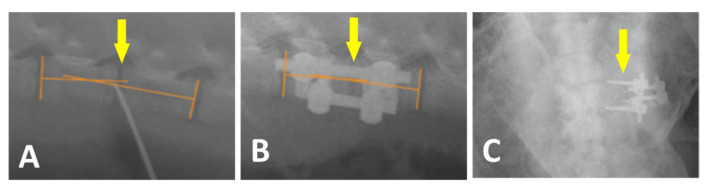
Plain X-ray radiographs of lateral views taken before (**A**) and after the surgery (**B**) Yellow arrows show the L2/L3 segment of implantation. Loosening and migration of the screws of the A2 group in 4 weeks after surgery. Plain X-ray radiographs of the antero-posterior view (**C**).

Plain radiographs were regularly repeated before and after the surgery as depicted in Figure 6A,B, respectively.

All 24 pigs recovered from the surgery without unusual events. One pig from group A2 showed loosening and migration of the screws observed on X-ray 4 weeks after surgery (Figure 6C). Regarding this animal, no clinical related manifestation was noted. No other hardware problems with the plate-screw-tube construct on regular X-ray examination were detected. No general intraoperative, early, or late postoperative complications were observed.

**Figure 7:**
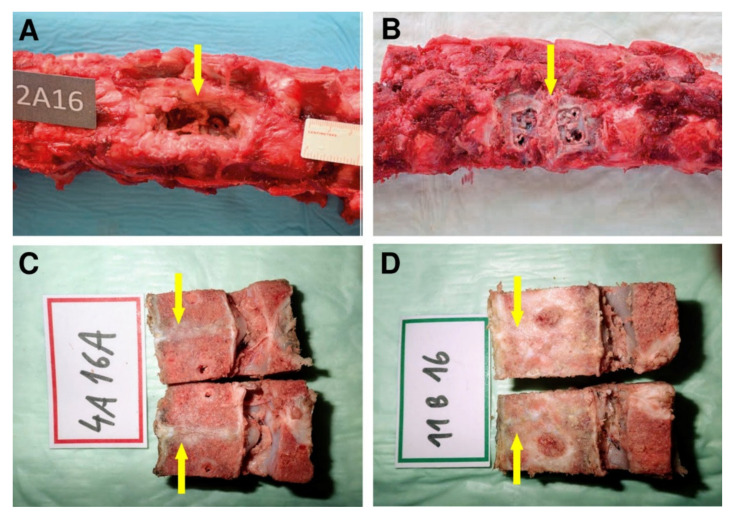
The autopsy photo shows a detail of the L2/3 segment of the spine of an animal from group A2 after 16 weeks of experiment (**A**). The arrow indicates the location after explantation of the osteosynthetic material with the presence of fibrous reaction. The excised segment of the spine of an animal from group B2, the arrow mark segment L2/3 after osteosynthetic explantation material, the fibrous reaction is not present, on the contrary, a partial bone overgrowth is expressed at the site after removal of anterior implant fixation plates (**B**). Fibrous reaction without osseous fusion was observed macroscopically as a white line (yellow arrow) at the site of bone autograft implantation of the cadaveric lumbar spine at L2/3 level of Group A (**C**). Macroscopically observed interbody fusion of group B2, 16 weeks after surgery (**D**). In contrast, the structure of the tissue of interbody fusion at the site of bioresorbable hybrid implant (BHI) implantation was solid (yellow arrow) like the surrounding bone with full osseous fusion as (**D**).

Subsequently, on the harvested spine segments, screw loosening in 3 animals in subgroup A1 and partial hardware loosening in 4 pigs in subgroup A2 was observed due to the fibrocartilage tissue mainly presented at the location after explantation of the osteosynthetic material (Figure 7A). That is an indirect sign of instability at the site of the intervertebral fusion. In group B, discrete screw loosening was noticed in 2 pigs, each in one subgroup. In the rest of group B2, the fixation plates were visually visible to the bone overgrowth and had to be loosened by cutting with a chisel (Figure 7B). Large fibrous reaction (connective tissue) without osseous fusion was observed macroscopically as a white line at the site of bone autograft implantation at the operated L2/3 level of Group A (Figure 7C). In contrast, the structure of the tissue of interbody fusion at the site of BHI implantation was solid like the surrounding bone with full osseous fusion (Figure 7D).

### 3.4. Micro-CT

**Figure 8:**
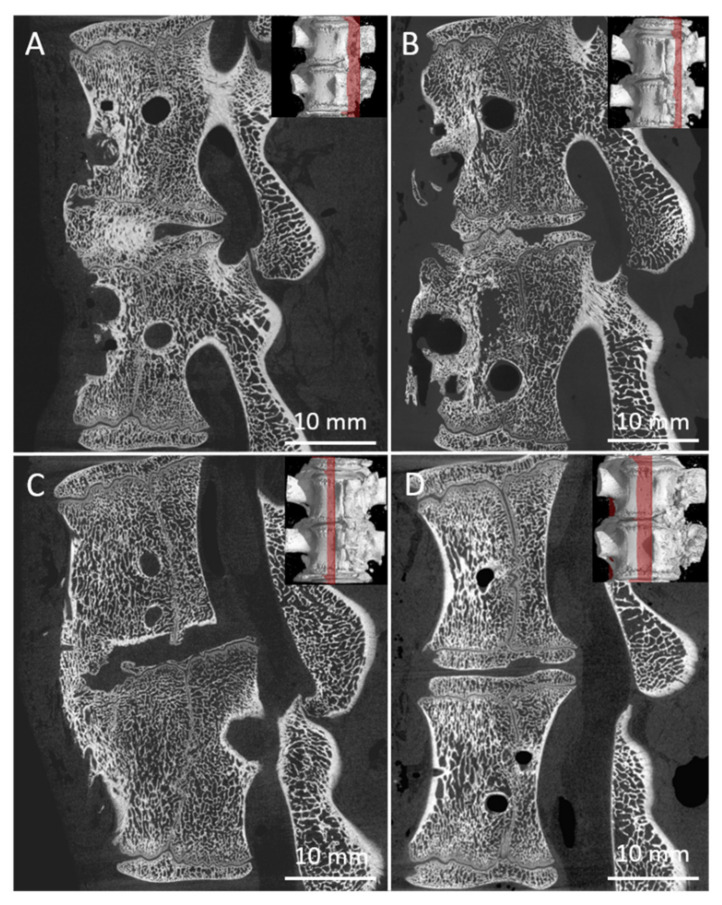
Examples of bone graft samples sorted into groups according to the quality of the fusion Grade I, II, III, IV, respectively. (**A**) fusion is visible and entirely joins the vertebra in the area of the bone graft, (**B**) fusion in the area of the bone graft is presented, but it is not completed, (**C**) fusion of the vertebras is visible but it is not in the area of bone graft, (**D**) no fusion observed.

The samples were divided into groups with different fusion quality according to CT appearance following [37]. The examples of Grade I (complete fusion), Grade II (partial fusion), Grade III (unipolar pseudarthrosis) and Grade IV (bipolar pseudarthrosis) are shown in Figure 8A–D. The complete absorption of the bone graft will be assigned to Grade IV.

**Figure 9:**
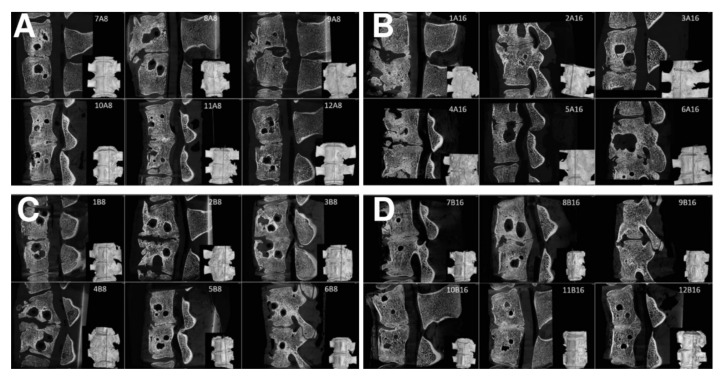
Sagittal micro-CT sections of the middle part of 6 samples from the Group A1 (**A**), Group A2 (**B**), Group B1 (**C**), and Group B2 (**D**), of the L2/3 section of pig spines. The plane of the section is recorded by a red line on the 3D reconstruction of the spine model.

There were 24 measurements performed in both groups, twelve for each group A (A1, Figure 9A; A2 Figure 9B) and B (B1, Figure 9C; B2, Figure 9D). All harvested spine samples from subgroups after 8 and 16 weeks were scanned immediately after pig euthanasia.

Samples were classified by the mentioned grading system, and the results of the analysis are in Table 1. Table 1 summarizes the results of the fusion assessments at the study endpoint after 8 and 16 weeks. After 8 weeks, we observed a similar distribution of fusion quality in both groups, without statistically significant difference. On the contrary, in group B, the results after 16 weeks showed the presence of fusion grade 1 or 2 in 6 specimens, while the number of samples in group A did not change.

**Figure 10:**
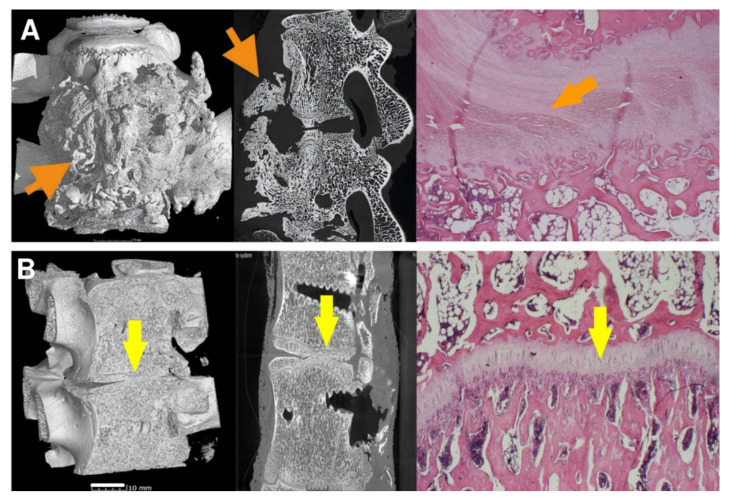
The vertebrae fusion of cadaveric lumbar spines is presented by micro-CT and histology images after 16 weeks after surgery for subgroups A2 (**A**) and B2 (**B**). Left pictures represent micro-CT 3D visualization, the middle images show micro-CT cross-section, and the right ones correspond to the histological images. On the micro-CT images, clearly visible nonunion outgrowth can be seen in Group A2 (autograft) (**A**, orange arrows), while the bone fusion of the intervertebral areas without connective tissue outgrowth marked with yellow arrows of group B2 (sample with bioresorbable hybrid implant (BHI)) is observed in figure (**B**). micro-CT images very well corresponded to the histological pictures, where the central fibrous cartilage (light pink) layer is in the middle of the bone tissue (**A**, right image), whereas endochondral ossification with new bone and without fibrous tissue was formed after BHI implantation (**B**, right image, yellow arrow). Histological decalcified samples were stained by hematoxylin-eosin to observe the nucleus of the cells (blue-violet), the cytoplasm (pink), collagen connective tissue (light pink), cartilage cells (dark blue), and muscle (red).

As can be seen from Table 1, the 8-week bone autograft group (subgroup A1) involved three samples, where the vertebras were surprisingly fused within the whole area of the bone graft. The fusion is steady and compact in micro-CT cross-sections. In one case, the bone graft was already fully absorbed exhibiting no fusion at all. However, after 16 weeks, the second group of bone grafts (subgroup A2) displayed a much lower quality of fusion, where the graft was completely absorbed in three cases. The vertebrae fused around the bone graft, and the fusion caused so-called non-union outgrowth around the whole vertebra’s body (Figure 10A). These large outgrowths have mostly affected the motion and bending of the spine. Moreover, intervertebral endplates are missing since they have been absorbed during the interaction with the bone graft. These disadvantages are widespread when a bone autograft is used.

As for the 8-week subgroup B1, where BHI was applied, the demonstrated results in Table 1 are very similar to subgroup A1. However, except for one fully absorbed sample, the fusion was mainly generated through/over the scaffold. Clearer are data from the 16-week subgroup B2, where fusions appear only between the bodies of the vertebra, and no large outgrowths were visible around the vertebrae in micro-CT images (Figure 10B). In addition, the intervertebral endplates were still found within the images, and in a few cases, intervertebral plate narrowing was observed.

### 3.5. Histology

**Figure 11:**
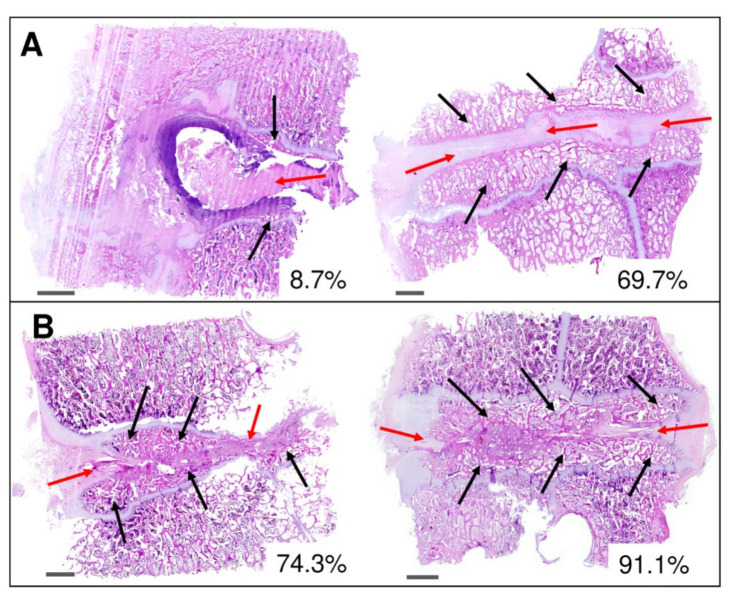
Histomorphological analysis of the intervertebral fusion area after 16 weeks in subgroup A2 (bone autograft) (**A**) and B2 (bioresorbable hybrid implant) (**B**). Red arrows depict fibrocartilage tissue in the area of the implementation site. Marked areas with black arrows represent newly formed trabecular bone tissue having visible bone marrow with trilineage hematopoiesis in the new bone formation. Selected images show low and high amount of bone fusion in %. Scalebar of 2 mm in each image.

To see the quality of the formed intervertebral fusions, the areas of mature bone were quantified from the histomorphological images using ImageJ software after 16 weeks from implantation as presented in Figure 11. The images showed significant differences between both subgroups A2 (Figure 11A) and B2 (Figure 11B). New bone formation values in subgroup A2 were very different between samples (from 8.7% to 69.%) due to the formation of fibrous nonunion tissue (Figure 11A, red arrows). Black arrows in Figure 11A depict the bone marrow with trilineage hematopoiesis in the new bone formation with a fibrocartilage tissue as a spacer between bone parts. In contrast, values of newly formed bone obtained after BHI implantation (Figure 11B, black arrows) with a small amount of fibrous tissue at the fusion edges (Figure 11B, red arrows) ranged from 74.3 to 91.1% proving the osteoconductive and osteoinductive ability of BHI material.

Using graphical evaluation with software ZEN 2.6 the area of newly formed bone was calculated and converted to mm^2^. Calculated areas of new bone were in the range of 44.1–48.1 and 58.3–72.5 mm^2^ for subgroups A2 and B2, respectively, proving a higher amount of newly formed trabecular bone in the fusion area treated with BHI material.

### 3.6. Biomechanics

The flexural stiffness in the extension of the lumbar spine cadavers, carried out on the specific Bionix Spine Kinematics equipment designed for spine loading, was evaluated for all subgroups (A1, A2, B1 and B2) as well as for the native untreated cadaveric spines (group N).

**Figure 12:**
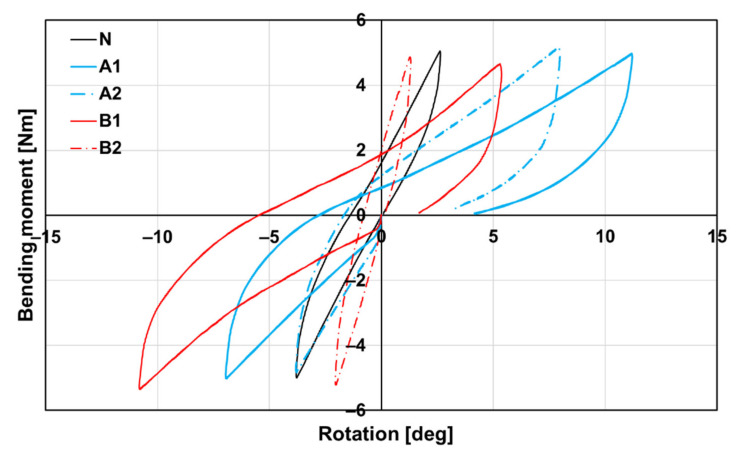
Dependence of bending moment on rotation according to groups A1, A2, B1, B2, and N (native bone) of spine cadavers. The steeper curve, the stiffer spine cadaver.

The graphic curves of all groups are shown in Figure 12. The differences in the slope of the curves in the extension region are evident from the above figure. When comparing the individual curves of subgroups A1, B1, and A2, B2, it can be said that the slope of the curve in the extension area is steeper after 16 weeks than after 8 weeks. This is indirect evidence that longer healing in the form of 16 weeks will achieve a better fusion of the connected parts of the spine and thus greater rigidity. After 16 weeks, it can also be stated that the curves in the extensive part of the course, especially of the subgroup B2 (red dashed line), begin to approach the curves recorded in group N (black line) in their shape.

**Figure 13:**
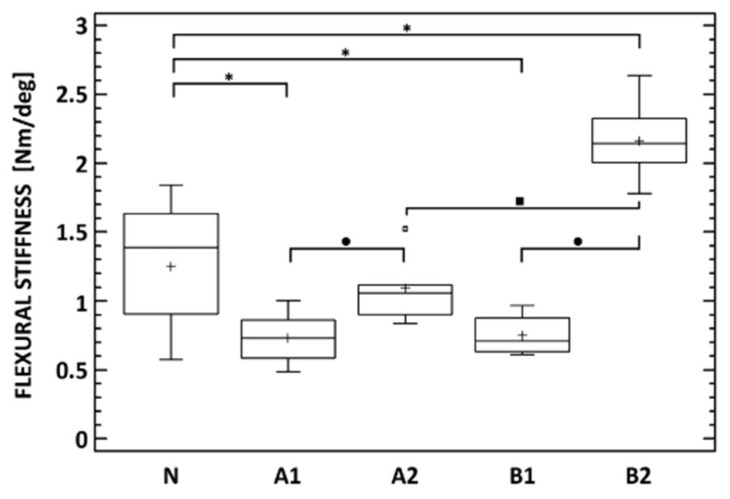
Flexural stiffness of native lumbar spine cadavers (N), specimens 8 (A1) and 16 (A2) weeks after lumbotomy and autologous Iliac crest bone graft insertion into the L2/3 intervertebral space, and specimens 8 (B1) and 16 (B2) weeks after lateral lumbar interbody fusion (L2/3) with the nanocomposite porous implant. * Denotes statistically significant differences determined between any of the treated groups and the native cadavers. • denotes statistically significant differences between the time points of each of the A and B groups. ▪ denotes statistically significant differences between the A and B group at each of the time points (Mann–Whitney test, *p* < 0.05).

The flexural stiffness in the extension of the lumbar spine cadavers was evaluated for all subgroups (A1, A2, B1, and B2) as well as for the native untreated cadaveric spines (group N). Eight weeks after the implantation of the bone graft (A1), the flexural stiffness of the spine cadavers remained significantly lower than the stiffness of native cadavers (N), as can be seen even in the case of subgroup B1. At week 8, the flexural stiffness of cadavers with BHI (B1) was comparable to the bone graft cadavers (A1) as we have already proved by micro-CT (Table 1). Very different situations occurred after 16 weeks of implantation, where the stiffness of cadavers with the bone graft (A2) was almost comparable to the native cadavers (N), while the flexural stiffness of cadavers with the BHI increased statistically significantly up to 150%. Eight weeks after implantation, there was no statistically significant difference between the stiffness of cadavers with the bone graft (A1) and the BHI (B1). Contrarily, after 16 weeks, the stiffness of cadavers with BHI (B2) was approximately two times higher (2.17 Nm/deg) than the stiffness of cadavers with the bone graft (A2). This increase demonstrates the effect of the biodegradable hybrid implant on the higher rate of intervertebral fusion compared with the bone graft (Figure 13). In the case of group A, an increase in the flexural stiffness of the spine was also demonstrated, which was not due to the quality of the fusion but rather to the formation of a perivertebral calcifying fibrous infiltrate, which was also demonstrated on micro-CT examination.

## 4. Discussion

Our newly developed implant represents a third-generation scaffold based on the fully degradable biphasic calcium phosphate matrix impregnated with a low concentration of nontoxic resorbable fibrous biopolymers (collagen and oxidized cellulose) thus mimicking the architecture and chemical composition and physical properties of the extracellular bone matrix and therefore simulating the osteoconductive characteristics of an autologous bone graft [38]. The stabilized fibroblast growth factor 2 FGF2-STAB^®^ added to the implant as an osteoinductive substance is supposed to accelerate osteogenesis, enhance the proliferation of the osteoblast cell line as well as induce angiogenesis [12]. The stability of natural growth factors is often limited to several hours due to protein aggregation (e.g., human FGF2 loses its activity after a 24 h incubation at 37 °C) [39]. Therefore, the applied dose is usually high and growth factors must be applied daily due to fast protein degradation [40,41]. To prolong the growth factor lifetime, FGF2 can be stabilized by, e.g., conjugation to heparin or heparin-like molecules, coacervation, chemical modifications, genetic engineering, physical barrier strategies, entrapment in hydrogels, microencapsulation, and adsorption [42]. Used FGF2-STAB^®^ is a human recombinant protein, a unique nine-point mutant of the low molecular weight isoform FGF2 with in vitro functional half time at 37 °C for more than 20 days [35]. The increased stability of FGF2 decreases the dependence of FGF2 on heparin, allowing lower doses in drug delivery carriers for in vivo studies [43]. Moreover, FGF2 can be stabilized via electrostatic ionic interactions by complexation with collagen type I scaffolds both in vitro and in vivo physiological conditions [44]. FGF2 bonded to collagen is both protected from the proteolytic environment and released in well-controlled manner from collagen matrices. The rate of FGF2 release can be influenced by several additives (e.g., heparin, gelatin, chitosan, cellulose) [45]. Moreover, collagen cross-linking with EDC/NHS system ensures sustained controlled release of FGF2 over 37 days [46].

Recently, we examined the FGF2-STAB^®^ release from collagen/chitosan scaffolds cross-linked with EDC/NHS system as the growth factor biological activity both in vitro on murine 3T3-A31 mouse fibroblast cells and in vivo on New Zealand white rabbits when supporting full-skin thickness regeneration [29]. Broad range of FGF2-STAB^®^ concentrations from 0 to 100 µg/mL was evaluated for cell metabolic activity, where lower concentrations from 0.01 up to 0.5 µg/mL showed enhanced cell metabolic activity and collagen expression compared to proteins with higher concentrations that may be beneficial for wound healing and tissue regeneration [47]. Our previous in vivo studies on a rabbit animal model revealed the superiority of scaffolds enriched with 0.1 µg/cm^2^ of FGF2-STAB^®^ in terms of scaffold bioresorption, minimal inflammation, and excellent granulation for skin tissue reconstruction. Gene expression proved the safety and positive effect of FGF2-STAB^®^ in collagen/chitosan porous scaffolds on skin regeneration, especially its dermal part.

This study aimed to confirm the safety and efficacy of long-term stable FGF2-STAB^®^ in vivo in combination with EDC/NHS cross-linked ceramic/collagen/oxidized cellulose implant within the lumbar interbody fusion creation. Prior to its an in vivo implantation, in vitro study verified the non-cytotoxicity of the BHI scaffold.

Based on the results of the three-week culture period, the BHI loaded with 0.1 µg/cm^2^ of FGF2-STAB^®^ was biocompatible and enabled cell adhesion and proliferation. The cells formed confluent layers already 24 h after seeding. As calculated from the SEM images, the BHI exhibited interconnected pores ranging from 100 to 1200 µm with an open porosity of 85% to assure that implants are osteoconductive and enabled ingrowth of the cells into the volume of the implants [48]. Moreover, osteoinductivity of the hybrid implant is guaranteed by the modification with FGF2-STAB^®^ since generally FGF2 signaling pathway activates the expression of genes associated with skeletal development [49].

Prior to the in vivo evaluation, the mechanical properties of BHI were tested. Modification of BCP ceramic matrix by low concentration of biopolymers and 0.1 µg/cm^2^ of FGF2-STAB^®^ exhibited negligible contribution to the mechanical properties of the whole ceramic implant, since e.g., the fully hydrated collagen possesses ultimate compressive strength and elastic modulus in compression two and three orders of magnitude lower than porous ceramic matrix, respectively [50]. Obtained values of mechanical properties are in accordance with the number of average values of both the total compression strength and elastic modulus of the vertebral body [51,52].

The proposed study was provided on the porcine spine of a conventional hybrid of domestic large white pigs that are relatively inexpensive and easy to manage with a short reproduction cycle. Anatomical and biomechanical evaluations of pig spines have shown comparable features with the human spine [53]. The similar vertebral size and anatomy enabled us to perform a standard fusion procedure using human implants and thus simulate real human spine surgery [54]. Based on animal species characteristics (young, rapidly growing pigs), shorter periods of follow-up were determined. Furthermore, similar time intervals to animal euthanasia are reported in other animal lumbar spinal fusion studies [55]. On the other hand, shorter time intervals to spinal fusion assessment might reduce the success rate of lumbar fusion [56].

Intervertebral fusion is standardly used in animal spine models. In our study, we decided on lumbar interbody fusion, which is a common surgical procedure used to treat severe degenerative disc disease [57]. Porcine anatomy and requirements for biomechanical testing (sufficient number of vertebral bodies above and below scaffold implantation) determined the optimal level (L2/3) of spinal fusion in our study. The L2/3 segment was stabilized using the lateral rod screw system without additional cage implementation. All pigs were divided into two study groups depending on the lateral lumbar interbody fusion (L2/3) provided either with implantation of an iliac crest bone autograft (group A, *n* = 12) or with a biomimetic BHI scaffold (group B, *n* = 12). Some studies have used a commercially available interbody PEEK cage as a control group [55,58]. However, the polymeric cage is not resorbable and there is a need of some autograft bone to fill its central part to support the bone fusion. Moreover, the cage stays in the implanted site after spine regeneration without its removal. Since we have provided specific biomechanical testing based on spine bending and torsion, we need to use some fully bioresorbable standardized materials for comparison with our BHI scaffold.

Macroscopic inspection of the explanted spines in group B elicited minimal inflammatory responses. On the contrary, in group A, a considerable fibrous inflammatory reaction around the bone autograft as well as hardware loosening were observed. These findings are in good correlation with the histological images as well as with the lower intervertebral stability confirmed by both micro-CT inspection and biomechanical studies.

Micro-CT scans taken postoperatively at week 8 showed similar fusion scores for both the BHI and autologous bone graft groups. In both groups, interbody fusion (grade I or II) was observed in four out of six animals [37]. Micro-CT scan analysis performed 16 weeks after surgery showed no trend toward higher fusion scores in group A. On the other hand, we achieved a statistically significant improvement of fusion rate in the BHI group. All 6 animals with the implanted BHI sacrificed 16 weeks after surgery exhibited interbody fusion (grade I or II). Ren et al. reported in their study a comparable fusion rate for an autologous tricortical iliac crest bone graft and multiaminoacid copolymer/nanohydroxyapatite/calcium sulphate 24 weeks after surgery [59]. In a study presented by Xu et al., a porous bioabsorbable interbody Mg-Zn alloy cage without any additives was applied, however, no acceptable results of intervertebral fusion were confirmed either on CT or biomechanical evaluation [60]. Abbah et al. presented unsatisfactory results of lumbar intervertebral fusion in the case of a BMP-free polycaprolactone/TCP scaffold compared to a bone autograft. In the scaffold with BMP group, they observed satisfactory intervertebral fusion after 9 months [61]. When compared to the BHI used in this study, we reached comparable results in a shorter period (4 months). Micro-CT was used as it is the most valuable imaging method for the evaluation of intervertebral bone healing. Although X-ray examination was performed and assessed, it was not sufficient for evaluation of bone fusion. Micro-CT results demonstrated superiority of the BHI scaffold over the tricortical bone graft. However, micro-CT, unlike plain radiography, does not underestimate vertebral segment stability [62,63]. This fact was supported by the biomechanical results, where a significant increase (up to 150%) in flexural stiffness was noticed after 16 weeks in group B compared to the native spine specimens. In contrast, the results of flexural stiffness in group A1 were significantly worse than the native bone specimens, but after 16 weeks the flexural stiffness had reached almost a level comparable with the native lumbar spine cadaver (no statistical difference). The biomechanical findings of flexural stiffness and moment correlated with the micro-CT results. Daentzer et al. tested a bioabsorbable interbody magnesium polymer cage and concluded inferior stiffness of the cage compared to a bone graft [64]. Similarly, Tang et al. reported lower values of maximum bending load and stiffness for a fast degradable citrated-based bone scaffold group in contrast with a bone graft [65]. Data from a study by Abbah et al. showed a significant increase in biomechanical stiffness in the experimental group with a bioabsorbable cage loaded with autogenous bone marrow stromal cell sheets compared to intact non-operated segments [66]. Sandhu et al. reached similar results in a titanium interbody fusion device filled with a rhBMP-2-collagen composite [67]. Yong et al. published double biomechanical stiffness obtained with a polycaprolactone based scaffold and rhBMP2 than with either scaffold alone or an autograft group. The results of our study with the FGF2-STAB^®^ enriched implant were comparable to the studies mentioned above. This fact supports the hypothesis of both its osteoinductive and osteoconductive potential [56].

In compliance with the biomechanics and micro-CT, the histomorphological results confirmed the progression of solid interbody fusion in group B between the 8th and 16th weeks. The trabecular newly formed bone in subgroup B2 involved from 74.3 to 91.1 %, whereas autograft Subgroup A2 exhibits only 8.7 to 69.7 %. This effect could be explained by the described macroscopic fibrous reaction with osseous outgrowth at the operated motion segment in group A2.

Shifts in anterior lumbar interbody fusion surgical approaches utilizing new interbody implants with anterior plating and screws have led to increased fusion rates in human medicine. According to a recent systematic review reported by Manzur that included 55 studies (5517 patients and 6303 fused vertebral levels), the average fusion rate following stand-alone was equal to 88.6% and newer zero-profile interbody implants caused a fusion rate of 89.2% [68]. Our fully bioresorbable hybrid implant provided a similar fusion rate as noticed above, accompanied by excellent biocompatibility and superior biomechanical properties of the fused spines.

## 5. Conclusions

In the proposed work, cell-free bioresorbable hybrid ceramic/polymer porous implant modified with collagen, oxidized cellulose and hyperstable FGF2-STAB^®^ protein was applied as a third-generation scaffold for spine regeneration. The spinal fusion was formed via bone tissue engineering dynamic process initiating osteoprogenitor cell migration followed by their proliferation, differentiation, and matrix formation along with bone ingrowth via osseointegration. Based on the experimental results, the application of the hybrid implant enriched with FGF2-STAB^®^ in experimental spinal fusion exhibited safety and considerable effectiveness with a significant osteoinductive effect in a porcine model even without the use of cell culturing. The effect was observed mainly on the biomechanical spine stiffness. Therefore, the bioresorbable hybrid implant loaded with FGF2-STAB^®^ can be stated as a promising safe candidate for bone tissue engineering, specifically for lumbar interbody fusion creation.

## Figures and Tables

**Table 1:** Samples of pig spines fused by autograft and bioresorbable hybrid implant (BHI) and their corresponding fusion grades.

	8 Weeks			16 Weeks		
Fusion Grade	Subgroup A1 (*n* = 6) Autograft	Subgroup B1 (*n* = 6) BHI	p	Subgroup A2 (*n* = 6) Autograft	Subgroup B2 (*n* = 6) BHI	p
I	3	2		1	5	
II	1	2	0.737	3	1	0.023 ^*^
III	1	1		2	0	
IV	1	1		0	0	

* Statistical significance of difference between groups was tested by the Mann–Whitney U test.

## Data Availability

The data presented in this study are available on request from the corresponding author.

## References

[1] Rajaee S.S., Bae H., Kanim L.E., Delamarter R.B. (2012). Spinal fusion in the United States. Spine.

[2] Chun D.S., Baker K.C., Hsu W.K. (2015). Lumbar pseudarthrosis: A review of current diagnosis and treatment. Neurosurg. Focus.

[3] Albrektsson T., Johansson C. (2002). Osteoinduction, osteoconduction and osseointegration. Use Bone Substit. Spine Surg..

[4] Seiler J.G., Johnson J. (2000). Iliac crest autogenous bone grafting: Donor site complications. J. South. Orthop. Assoc..

[5] Qu H., Fu H., Han Z., Sun Y. (2019). Biomaterials for bone tissue engineering scaffolds: A review. RSC Adv..

[6] Einhorn T.A. (1998). The cell and molecular biology of fracture healing. Clin. Orthop. Relat. Res..

[7] El Bialy I., Jiskoot W., Nejadnik M.R. (2017). Formulation, Delivery and Stability of Bone Morphogenetic Proteins for Effective Bone Regeneration. Pharm. Res..

[8] Ye F., Zeng Z., Wang J., Liu H., Zheng Z. (2017). Comparison of the use of rhBMP-7 versus iliac crest autograft in single-level lumbar fusion: A meta-analysis of randomized controlled trials. J. Bone Miner. Metab..

[9] Cottrill E., Ahmed A.K., Lessing N., Pennington Z., Ishida W., Perdomo-Pantoja A., Lo S.-F., Howell E., Holmes C., Goodwin C.R. (2019). Investigational growth factors utilized in animal models of spinal fusion: Systematic review. World J. Orthop..

[10] Coffin J.D., Homer-Bouthiette C., Hurley M.M. (2018). Fibroblast Growth Factor 2 and Its Receptors in Bone Biology and Disease. J. Endocr. Soc..

[11] Inoue G., Uchida K., Matsushita O., Fujimaki H., Saito W., Miyagi M., Sekiguchi H., Nishi N., Ohtori S., Yogoro M. (2017). Effect of freeze-dried allograft bone with human basic fibroblast growth factor containing a collagen-binding domain from clostridium histolyticum collagenase on bone formation after lumbar poster-olateral fusion surgery in rats. Spine.

[12] Charoenlarp P., Rajendran A.K., Iseki S. (2017). Role of fibroblast growth factors in bone regeneration. Inflamm. Regen..

[13] Buchtova M., Chaloupkova R., Zakrzewska M., Vesela I., Cela P., Barathova J., Gudernova I., Zajickova R., Trantirek L., Martin J. (2015). Instability restricts signaling of multiple fibroblast growth factors. Cell. Mol. Life Sci..

[14] Enantis (2017). Stable Fibroblast Growth Factor 2 FGF2-STAB®.

[15] Nečas A., Proks P., Urbanová L., Srnec R., Stehlík L., Crha M., Raušer P., Plánka L., Janovec J., Dvořák M. (2010). Healing of Large Segmental Bone Defect after Implantation of Autogenous Cancellous Bone Graft in Comparison to Hydroxyapatite and 0.5% Collagen Scaffold Combined with Mesenchymal Stem Cells. Acta Veter. Brno.

[16] Prosecka E., Rampichova M., Vojtová L., Tvrdik D., Melčáková Š., Juhasova J., Plencner M., Jakubová R., Necas A., Klepáček J. (2011). Optimized conditions for mesenchymal stem cells to differentiate into osteoblasts on a collagen/hydroxyapatite matrix. J. Biomed. Mater. Res. Part A.

[17] Plánka L., Nečas A., Crha M., Proks P., Vojtova L., Gal P. (2011). Treatment of a bone bridge by transplantation of mesenchymal stem cells and chon-drocytes in a composite scaffold in pigs. Experimental study. Acta Chir. Orthop. Traumatol. Cech..

[18] Nečas A., Plánka L., Srnec R., Crha M., Hlučilová J., Klíma J., Starý D., Křen L., Amler E., Vojtova L. (2010). Quality of newly formed cartilaginous tissue in defects of articular surface after transplantation of mesenchymal stem cells in a composite scaffold based on collagen i with chitosan micro- and nanofibres. Physiol. Res..

[19] Karageorgiou V., Kaplan D. (2005). Porosity of 3D biomaterial scaffolds and osteogenesis. Biomaterials.

[20] Wang M. (2006). Composite Scaffolds for Bone Tissue Engineering. Am. J. Biochem. Biotechnol..

[21] Sukhodub L., Moseke C., Sulkio-Cleff B., Maleev V., Semenov M., Bereznyak E., Bolbukh T. (2004). Collagen–hydroxyapatite–water interactions investigated by XRD, piezogravimetry, infrared and Raman spectroscopy. J. Mol. Struct..

[22] Sachlos E., Gotora D., Czernuszka J.T. (2006). Collagen scaffolds reinforced with biomimetic composite nano-sized carbonate-substituted hydroxyapatite crystals and shaped by rapid prototyping to contain internal microchannels. Tissue Eng..

[23] Prosecka E., Rampichova M., Litvinec A., Tonar Z., Kralickova M., Vojtová L., Kochova P., Plencner M., Buzgo M., Mickova A. (2015). Collagen/hydroxyapatite scaffold enriched with polycaprolactone nanofibers, thrombocyte-rich solution and mesenchymal stem cells promotes regeneration in large bone defect in vivo. J. Biomed. Mater. Res. Part A.

[24] Veillette C.J., McKee M.D. (2007). Growth factors—BMPs, DBMs, and buffy coat products: Are there any proven differences amongst them?. Injury.

[25] Babrnáková J., Pavliňáková V., Brtníková J., Sedláček P., Prosecká E., Rampichová M., Filová E., Hearnden V., Vojtová L. (2019). Synergistic effect of bovine platelet lysate and various polysaccharides on the biological properties of collagen-based scaffolds for tissue engineering: Scaffold preparation, chemo-physical characterization, in vitro and ex ovo evaluation. Mater. Sci. Eng. C.

[26] Ong S.-Y., Wu J., Moochhala S.M., Tan M.-H., Lu J. (2008). Development of a chitosan-based wound dressing with improved hemostatic and antimicrobial properties. Biomaterials.

[27] Novotna K., Havelka P., Sopuch T., Kolarova K., Vosmanska V., Lisa V., Svorcik V., Bacakova L. (2013). Cellulose-based materials as scaffolds for tissue engineering. Cellulose.

[28] Hosoya T., Bacher M., Potthast A., Elder T., Rosenau T. (2018). Insights into degradation pathways of oxidized anhydroglucose units in cellulose by β-alkoxy-elimination: A combined theoretical and experimental approach. Cellulose.

[29] Vojtová L., Pavliňáková V., Muchová J., Kacvinská K., Brtníková J., Knoz M., Lipový B., Faldyna M., Göpfert E., Holoubek J. (2021). Healing and Angiogenic Properties of Collagen/Chitosan Scaffolds Enriched with Hyperstable FGF2-STAB^®^ Protein: In Vitro, Ex Ovo and In Vivo Comprehensive Evaluation. Biomedicines.

[30] Muchová J., Hearnden V., Michlovská L., Vištejnová L., Zavaďáková A., Šmerková K., Kočiová S., Adam V., Kopel P., Vojtová L. (2021). Mutual influence of selenium nanoparticles and FGF2-STAB® on biocompatible properties of collagen/chitosan 3D scaffolds: In vitro and ex ovo evaluation. J. Nanobiotechnology.

[31] Dong C., Lv Y. (2016). Application of collagen scaffold in tissue engineering: Recent advances and new perspectives. Polymer.

[32] Šťastný P., Sedlacek R., Suchý T., Lukasova V., Rampichova M., Trunec M. (2019). Structure degradation and strength changes of sintered calcium phosphate bone scaffolds with different phase structures during simulated biodegradation in vitro. Mater. Sci. Eng. C.

[33] Šťastný P., Chlup Z., Kalasova D., Zikmund T., Kaiser J., Trunec M. (2018). Epoxy-based gelcasting of machinable hydroxyapatite foams for medical applications. J. Am. Ceram. Soc..

[34] Sloviková A., Vojtová L., Jančař J. (2008). Preparation and modification of collagen-based porous scaffold for tissue engineering. Chem. Pap..

[35] Dvorak P., Bednar D., Vanacek P., Balek L., Eiselleova L., Stepankova V., Sebestova E., Bosakova M., Konecna Z., Mazurenko S. (2018). Computer-assisted engineering of hyperstable fibroblast growth factor 2. Biotechnol. Bioeng..

[36] Schneider C.A., Rasband W.S., Eliceiri K.W. (2012). NIH Image to ImageJ: 25 years of image analysis. Nat. Methods.

[37] Tan G.H., Goss B., Thorpe P.J., Williams R.P. (2007). CT-based classification of long spinal allograft fusion. Eur. Spine J..

[38] Scholz M., Schleicher P., Eindorf T., Friedersdorff F., Gelinsky M., König U., Sewing A., Haas N., Kandziora F. (2010). Cages augmented with mineralized collagen and platelet-rich plasma as an osteoconductive/inductive combination for interbody fusion. Spine.

[39] Chen G., Gulbranson D.R., Yu P., Hou Z., Thomson J.A. (2011). Thermal stability of fibroblast growth factor protein is a determinant factor in regulating self-renewal, differentiation, and reprogramming in human pluripotent stem cells. Stem Cells.

[40] Andreopoulos F.M., Persaud I. (2006). Delivery of basic fibroblast growth factor (bFGF) from photoresponsive hydrogel scaffolds. Biomaterials.

[41] Cai S., Liu Y., Shu X.Z., Prestwich G.D. (2005). Injectable glycosaminoglycan hydrogels for controlled release of human basic fibroblast growth factor. Biomaterials.

[42] Benington L., Rajan G., Locher C., Lim L.Y. (2020). Fibroblast Growth Factor 2—A Review of Stabilisation Approaches for Clinical Applications. Pharmaceutics.

[43] Koledova Z., Sumbal J., Rabata A., De La Bourdonnaye G., Chaloupkova R., Hrdlickova B., Damborsky J., Stepankova V. (2019). Fibroblast growth factor 2 protein stability provides decreased dependence on heparin for induction of FGFR signaling and alters ERK signaling dynamics. Front. Cell Dev. Biol..

[44] Kanematsu A., Marui A., Yamamoto S., Ozeki M., Hirano Y., Yamamoto M., Ogawa O., Komeda M., Tabata Y. (2004). Type I collagen can function as a reservoir of basic fibroblast growth factor. J. Control. Release.

[45] Munisso M.C., Morimoto N., Notodihardjo S.C., Mitsui T., Kakudo N., Kusumoto K. (2019). Collagen/Gelatin Sponges (CGSs) Provide Both Protection and Release of bFGF: An In Vitro Study. BioMed Res. Int..

[46] Wu J.M., Xu Y.Y., Li Z.H., Yuan X.Y., Wang P.F., Zhang X.Z., Liu Y.Q., Guan J., Guo Y., Li R.X. (2011). Heparin-functionalized collagen matrices with controlled release of basic fibroblast growth factor. J. Mater. Sci. Mater. Med..

[47] Ludwig T.E., Levenstein M.E., Jones J.M., Berggren W.T., Mitchen E.R., Frane J.L., Crandall L.J., A Daigh C., Conard K.R., Piekarczyk M.S. (2006). Derivation of human embryonic stem cells in defined conditions. Nat. Biotechnol..

[48] Oh S.H., Park I.K., Kim J.M., Lee J.H. (2007). In vitro and in vivo characteristics of PCL scaffolds with pore size gradient fabricated by a centrifugation method. Biomaterials.

[49] Marie P. (2003). Fibroblast growth factor signaling controlling osteoblast differentiation. Gene.

[50] Suchý T., Šupová M., Bartoš M., Sedláček R., Piola M., Soncini M., Fiore G.B., Sauerova P., Kalbacova M.H. (2018). Dry versus hydrated collagen scaffolds: Are dry states representative of hydrated states?. J. Mater. Sci. Mater. Med..

[51] Mosekilde L., Mosekilde L. (1986). Normal vertebral body size and compressive strength: Relations to age and to vertebral and iliac trabecular bone compressive strength. Bone.

[52] De Faria S.P. (2015). Biomechanical Analysis of the Human Lumbar Spine—An Experimental and Computational Approach.

[53] Busscher I., van der Veen A.J., van Dieën J.H., Kingma I., Verkerke G.J., Veldhuizen A.G. (2010). In vitro biomechanical characteristics of the spine. Spine.

[54] Lee J.H., Nam Y., Lee J.-H. (2017). Animal models of orthopedic research: A spinal fusion model. J. Korean Orthop. Assoc..

[55] McGilvray K.C., Waldorff E.I., Easley J., Seim H.B., Zhang N., Linovitz R.J., Ryaby J.T., Puttlitz C.M. (2017). Evaluation of a polyetheretherketone (PEEK) titanium composite interbody spacer in an ovine lumbar interbody fusion model: Biomechanical, microcomputed tomographic, and histologic analyses. Spine J..

[56] Yong M.R., Saifzadeh S., Askin G.N., Labrom R.D., Hutmacher D.W., Adam C.J. (2013). Biological performance of a polycaprolactone-based scaffold plus recombinant human morphogenetic protein-2 (rhBMP-2) in an ovine thoracic interbody fusion model. Eur. Spine J..

[57] Chau A.M.T., Xu L.L., Wong J.H.-Y., Mobbs R.J. (2013). Current status of bone graft options for anterior interbody fusion of the cervical and lumbar spine. Neurosurg. Rev..

[58] Sherman B.P., Lindley E.M., Turner A.S., Iii H.B.S., Benedict J., Burger E.L., Patel V.V. (2010). Evaluation of ABM/P-15 versus autogenous bone in an ovine lumbar interbody fusion model. Eur. Spine J..

[59] Ren C., Song Y., Xue Y., Yang X., Zhou C. (2017). Evaluation of bioabsorbable multiamino acid copolymer/nanohydroxyapatite/calcium sulfate cage in a goat spine model. World Neurosurg..

[60] Xu H., Zhang F., Wang H., Geng F., Shao M., Xu S., Xia X., Ma X., Lu F., Jiang J. (2018). Evaluation of a Porous Bioabsorbable Interbody Mg-Zn Alloy Cage in a Goat Cervical Spine Model. BioMed Res. Int..

[61] Abbah S.A., Lam C.X., Ramruttun K.A., Goh J.C., Wong H.-K. (2011). Autogenous bone marrow stromal cell sheets-loaded mpcl/tcp scaffolds induced osteogenesis in a porcine model of spinal interbody fusion. Tissue Eng. Part A.

[62] Dewan A.K., Dewan R.A., Calderon N., Fuentes A., Lazard Z., Davis A.R., Heggeness M., Hipp J.A., Olmsted-Davis E.A. (2010). Assessing mechanical integrity of spinal fusion by in situ endochondral oste-oinduction in the murine model. J. Orthop. Surg. Res..

[63] Kroeze R.J., Smit T.H., Vergroesen P.-P., Bank R.A., Stoop R., Van Rietbergen B., Van Royen B.J., Helder M.N. (2014). Spinal fusion using adipose stem cells seeded on a radiolucent cage filler: A feasibility study of a single surgical procedure in goats. Eur. Spine J..

[64] Daentzer D., Willbold E., Kalla K., Bartsch I., Masalha W., Hallbaum M., Hurschler C., Kauth T., Kaltbeitzel D., Hopmann C. (2014). Bioabsorbable interbody magnesium-polymer cage. Spine.

[65] Tang J., Guo J., Li Z., Yang C., Xie D., Chen J., Li S., Li S., Kim G.B., Bai X. (2015). A fast degradable citrate-based bone scaffold promotes spinal fusion. J. Mater. Chem. B.

[66] Abbah S.A., Lam C.X., Ramruttun A.K., Goh J.C., Wong H.-K. (2011). Fusion performance of low-dose recombinant human bone morphogenetic protein 2 and bone marrow-derived multipotent stromal cells in biodegradable scaffolds. Spine.

[67] Sandhu H.S., Toth J.M., Diwan A., Seim H.B., Kanim L.E., Kabo J.M., Turner A.S. (2002). Histologic evaluation of the efficacy of rhbmp-2 compared with autograft bone in sheep spinal anterior interbody fusion. Spine.

[68] Manzur M., Virk S.S., Jivanelli B., Vaishnav A.S., McAnany S.J., Albert T.J., Iyer S., Gang C.H., Qureshi S. (2019). The rate of fusion for stand-alone anterior lumbar interbody fusion: A systematic review. Spine J..

